# Cathepsin L Helps to Defend Mice from Infection with Influenza A

**DOI:** 10.1371/journal.pone.0164501

**Published:** 2016-10-07

**Authors:** Xiang Xu, John R. Greenland, Jeffrey E. Gotts, Michael A. Matthay, George H. Caughey

**Affiliations:** 1 Department of Medicine, University of California at San Francisco, San Francisco, California, United States of America; 2 Veterans Affairs Medical Center, San Francisco, California, United States of America; 3 Cardiovascular Research Institute, University of California at San Francisco, San Francisco, California, United States of America; Deutsches Primatenzentrum GmbH - Leibniz-Institut fur Primatenforschung, GERMANY

## Abstract

Host-derived proteases can augment or help to clear infections. This dichotomy is exemplified by cathepsin L (CTSL), which helps Hendra virus and SARS coronavirus to invade cells, but is essential for survival in mice with mycoplasma pneumonia. The present study tested the hypothesis that CTSL protects mice from serious consequences of infection by the orthomyxovirus influenza A, which is thought to be activated by host-supplied proteases other than CTSL. *Ctsl*^-/-^ mice infected with influenza A/Puerto Rico/8/34(H1N1) had larger lung viral loads and higher mortality than infected *Ctsl*^+/+^ mice. Lung inflammation in surviving infected mice peaked 14 days after initial infection, accompanied marked focal distal airway bronchiolization and epithelial metaplasia followed by desquamation and fibrotic interstitial remodeling, and persisted for at least 6 weeks. Most deaths occurred during the second week of infection in both groups of mice. In contrast to mycoplasma pneumonia, infiltrating cells were predominantly mononuclear rather than polymorphonuclear. The histopathology of lung inflammation and remodeling in survivors was similar in *Ctsl*^-/-^ and *Ctsl*^+/+^ mice, although *Ctsl*^+/+^ mice cleared immunoreactive virus sooner. Furthermore, *Ctsl*^-/-^ mice had profound deficits in CD4+ lymphocytes before and after infection and weaker production of pathogen-specific IgG. Thus, CTSL appears to support innate as well as adaptive responses, which confer a survival advantage on mice infected with the orthomyxovirus influenza A.

## Introduction

Cathepsins are a group of proteolytic enzymes, classically lysosomal in origin, that vary in class, pH optimum, target specificity, inhibition and function. They are nearly universally expressed by eukaryotic organisms, in which they regulate a range of physiological processes, befitting their varied attributes and target preferences [1]. A subset of these proteases—the cysteinyl cathepsins—was characterized originally as being lysosomal in location and function. Later work uncovered additional roles in intracellular and extracellular locations [2]. Some of these enzymes cleave specific substrates involved in antigen presentation, collagen turnover in bone and cartilage, processing of endogenous bioactive peptides, and leukocyte penetration of cellular monolayers and matrix [1–5]. The widely expressed cysteine protease cathepsin L (CTSL) is implicated in several types of pathology and is a potential pharmaceutical target for inhibition to control inflammation and counterproductive immune responses. For example, by cleaving viral proteins, CTSL helps Ebola, Hendra and Severe Acute Respiratory Syndrome (SARS) viruses infect host cells, thereby advancing these infections [6–8]. On the other hand, in epithelial cells of the thymic cortex, CTSL plays a key role in removing the placeholder class II-associated invariant chain peptide, which is important for positive selection of CD4+ T cells that bind to MHC proteins on other cells. This role in T cell maturation can influence MHC-restricted presentation of and response to extracellular antigens, such as those from bacteria [9].

A previous study from this laboratory revealed that CTSL is critical for defense against lung infection by *Mycoplasma pulmonis*, which is an extracellular pathogen [10]. For many other respiratory pathogens, including the orthomyxovirus influenza A, there are no published data to indicate whether CTSL promotes infection, as suggested for some viral pathogens, or mitigates it, as in mycoplasma infection.

Influenza A viruses are among the most common and pathologically significant causes of human respiratory infections. Influenza epidemics are associated with major spikes in mortality every year worldwide, and the fortunately rare but more severe pandemics caused by new forms of virus can be catastrophic. Influenza A can be prevented by vaccines, which, however, may be partially or completely ineffective if they lack major epitopes of strains that are prevalent in a given year. Antiviral treatments available for active infections caused by some strains of influenza A can reduce influenza severity and duration but often have limited efficacy. Among potential alternative approaches, protease inhibition is a potential strategy because cleavage of influenza virus surface hemagglutinin by host cell proteases is a prerequisite for infectivity and pathogenicity [11]. Several candidate influenza A-activating proteases have been identified [12, 13], including the host epithelial protease TMPRSS2, which is influenza A strain-selective [14–16]. Most identified influenza virus-activating proteases are extracellular serine proteases of tryptic specificity. However, recent evidence suggests that calpains, which are non-cathepsin, neutral, cytosolic cysteinyl proteinases, promote influenza in mice, and that a calpain inhibitor reduces influenza-associated mortality [17]. The extent to which calpain effects are mediated by cleavage-activation of virus is not yet clear. Like calpains, CTSL is a cysteinyl proteinase but differs in having an acidic pH optimum that optimizes activity in lysosomes. CTSL cleaves some targets at tryptic sites, but also at non-tryptic sites, particularly in proteins co-localizing in lysosomes. Outside of the cell, CTSL activity is limited at the neutral to alkaline pH typical of extracellular environments and is opposed by extracellular inhibitors, the cystatins [1, 2]. On these grounds, we hypothesized that CTSL is less likely to be a major in vivo activator of influenza A than to support host defense against infection. This hypothesis predicts that influenza will be more severe in mice lacking CTSL. The present work tested this prediction by comparing immune and histopathological responses in virus-naïve wild-type and CTSL-deficient mice.

## Materials and Methods

### Infection with Influenza A

This study used male and female *Ctsl*^-/-^ mice described originally by Nakagawa and colleagues [9], received at the University of California at San Francisco in a C57BL/6/129S background from Shi and colleagues [18], then backcrossed 10 or more generations into the C57BL/6 background. Male and female *Ctsl*^+/+^ C57BL/6 mice served as wild-type controls. The study was conducted in strict accordance with the recommendations in the Guide for the Care and Use of Laboratory Animals of the National Institutes of Health. The protocol (number AN104626) was approved by the University of California at San Francisco’s Institutional Animal Care and Use Committee. Mice were 8–12 weeks of age at the beginning of the study, when they were anesthetized and inoculated intranasally with 50 μl of PBS containing 400 focus-forming units (FFU) of influenza A/H1N1/Puerto Rico/8/34 [19] or with 50 μl of sterile PBS alone (sham infection). Mice were weighed every two days. Animals determined to have lost > 25% of body weight or to be moribund (as indicated by increased respiratory rate and inability to ambulate) were euthanized.

### Histopathological Detection of Pneumonia and Fibrosis

After infection, mice were sacrificed at intervals up to 42 days. Pneumonia severity was graded histopathologically as described by Beck and colleagues in mice infected with influenza A H3N2 [20]. Briefly, scores were assigned according to degree of tissue infiltration with inflammatory cells: 0, no inflammation; 1, mild influx of inflammatory cells with cuffing around vessels; 2, increased inflammation involving 25–50% of lung; 3, severe inflammation involving 50–75% of lung; and 4, > 75% of lung infiltrated with inflammatory cells. To detect lung and airway fibrosis, additional tissue sections were stained with Masson’s trichrome (American Mastertech, Lodi, CA, USA) after 14 and 42 days of infection.

### Lung Viral Burden

Lung FFUs were measured 3 days after viral inoculation. Activatable virus was assayed by application of stock virus or extracts of whole lung (homogenized in 4 ml of PBS) to the apical surface of Madin-Darby canine kidney (MDCK) cells (American Type Culture Collection, Manassus, VA, USA) grown to confluence in individual wells of 96-well plates. After 1 h, samples were decanted and replaced with serum-free medium containing 100 μg/ml of L-(tosylamido-2-phenyl) ethyl chloromethyl ketone-treated bovine trypsin to activate any uncleaved virus. Sixteen hours later, cells were fixed in 100% cold methanol. Foci of infection were detected immunocytochemically by incubation with polyclonal 1:1,500 goat anti-influenza A IgG (AB1074; EMD Millipore, Billerica, MA, USA) followed by amplification and detection using biotinylated rabbit anti-goat IgG (Santa Cruz Biotechnology, Dallas, TX, USA) and Histostain-SP (streptavidin-peroxidase) with diaminobenzidine as chromogen (Invitrogen, Camarillo, CA, USA). Serial dilutions of samples were processed in triplicate. Lung FFUs were estimated from counts in wells in which dilutions yielded 30–100 discrete foci.

### Analysis of Bronchoalveolar Lavage (BAL) Fluid

To obtain BAL fluid for analysis, a sterile, 22-gauge catheter was inserted into exposed tracheal lumen of anesthetized mice. BAL fluid collected from one 0.8-ml aliquot of PBS per mouse was placed on ice and centrifuged. Supernatants were stored at –20°C; cell pellets were resuspended in PBS. Living cells were counted by staining with 0.4% trypan blue. Differential cell counts were obtained from cytospun cells stained with Diff-quik (American Scientific Products, McGaw Park, IL, USA). For further typing by analysis of surface protein expression, BAL cells were transferred into FACS buffer (PBS, 0.5% albumin, 0.1% sodium azide). Cells were divided and stained with either FITC anti-Gr1 (#RB6-865, UCSF Antibody Core Facility, San Francisco, CA, USA), PE anti-CD86 (553692, BD Pharmingen, San Diego, CA, USA), PE-Cy7 anti-CD11c (25-0114-82, eBioscience, San Diego, CA, USA), and APC anti-F4/89 (17-4801-82, eBioscience), or with FITC anti-CD3 (1530–02, Southern Biotech, Birmingham, AL, USA), PE anti-CD4 (553049, BD Pharmingen), PE-Cy7 anti-CD8 (552877, BD Pharmingen), APC anti NK1.1 (130-102-350, Miltenyi Biotec, San Diego, CA, USA), and APC-Cy7 anti-CD45 (557659, BD Pharmingen). Cells were stained for 30 min on ice, washed and then fixed in 2% paraformaldehyde (Sigma-Aldrich, St. Louis, MO, USA) before analyzing using a Navios flow cytometer (Beckman Coulter, Brea, CA, USA).

### Analysis of Splenocytes

Spleens excised from euthanized mice were placed into PBS. Splenocytes were extracted by passing spleens though wire mesh into wash media containing RPMI with 2% fetal calf serum, penicillin and streptomycin. The cells were then filtered through 70-micron nylon mesh (Elko, Miami Gardens, FL, USA) and transferred to FACS buffer, labeled with fluorescent probes and subjected to flow cytometry as described for cells obtained from BAL fluid.

### Immunohistochemical Studies

Lungs were fixed in 4% neutral formaldehyde, dehydrated in a series of graded ethanol solutions, and embedded in paraffin. For immunohistochemical detection of virus-infected cells and tissues, 5-μm sections were incubated overnight at 4°C with polyclonal goat anti-influenza A (AB1074; EMD Millipore) and then for 1 h with rabbit anti-goat biotin (Santa Cruz Biotechnology), followed by amplification and visualization with Histostain-SP (diaminobenzidine-based; Invitrogen), and counterstaining with hematoxylin. Immunoreactivity was quantitatively compared in infected *Ctsl*^*-/-*^ and *Ctsl*^*++*^ lung sections using the IHC Toolbox plugin for ImageJ software (National Institutes of Health), which supports automated, selective statistical detection of the brown color generated by diaminobenzidine staining in random microscopic fields of tissue sections.

### Measurement of Influenza A H1N1-specific Antibodies

Individual wells of immunoassay plates were coated with influenza A H1N1 antigen (2 x 10^5^ FFU/well in 50-mM carbonate buffer, pH 9.6) and incubated overnight at 4°C. Wells then were incubated for 2 h with 1% BSA/PBS to block non-specific binding. Serum from experimental animals was diluted 1:20 (to detect IgG1 and IgG2a) or 1:10 (to detect IgA, IgE and IgM) and then further diluted in serial fashion in PBS/0.05% Tween-20/0.5% BSA. 10–20 μl of diluted serum in each well were mixed with 50 μl of biotinylated anti-mouse IgG1, IgG2a, IgA, IgE or IgM solution (BD Pharmingen) and incubated overnight, followed by addition of 50 μl of alkaline phosphatase-conjugated streptavidin (1:3000; Jackson ImmunoResearch, West Grove, PA, USA). Alkaline phosphatase activity remaining bound after washing was detected using phosphatase substrate (4-nitrophenyl phosphate; Sigma-Aldrich) with spectrophotometric monitoring of cleaved substrate at 405 nm.

### Measurement of Cytokines and Chemokines

Levels in BAL fluid and lung extracts were assayed with ELISA kits for IFN-α (PBL Assay Science, Piscataway, NJ, USA), IFN-γ, TNF-α, monocyte chemoattractant protein 1 (MCP-1; eBioscience), macrophage inflammatory protein 2 (MIP-2; R&D Systems, Minneapolis, MN, USA) and IL-6 (BD Pharmingen).

### Statistical Analysis

Data were compared by *t*-test, log-rank test (for survival data), or one-way ANOVA (with Bonferroni’s test for pairs of groups). *P* < 0.05 was considered to be significant.

## Results

### Weight Loss and Death after Infection

**Fig 1:**
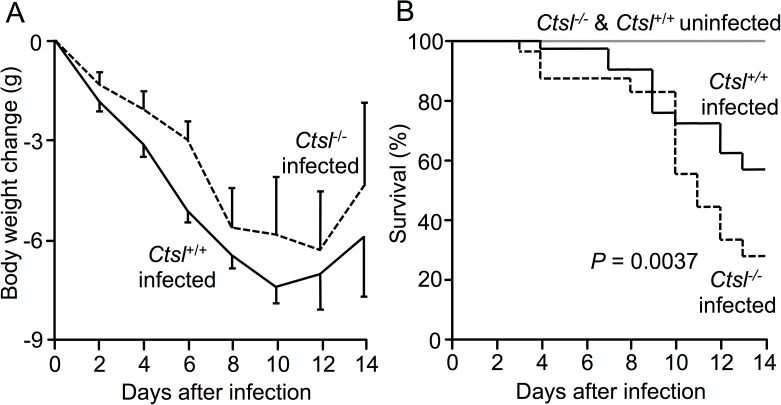
Change in body weight and survival in infected mice. (*A)* Weight loss was similar in surviving influenza A-infected *Ctsl*^-/-^ and *Ctsl*^+/+^ mice. Data shown are means ± SEM, with no significant difference between genotypes (*t*-test). (*B)* Survival in infected *Ctsl*^+/+^ mice over the same 14-day interval was better than in infected *Ctsl*^-/-^ mice (*P* = 0.0037 by log-rank test). No deaths occurred in sham-infected mice of either genotype. *N* = 5, 5, 23 and 32 in sham-infected *Ctsl*^-/-^, sham-infected *Ctsl*^+/+^, infected *Ctsl*^-/-^, and infected *Ctsl*^+/+^ mice, respectively.

To investigate overall severity of influenza, body weight and mortality were compared in age-matched *Ctsl*^-/-^ and *Ctsl*^+/+^ mice. Both types of mice lost weight after infection (Fig 1A). Survivors among infected mice in both groups began to regain weight after approximately 12 days. No significant differences in weight changes were found between the two types of infected mice. However, mortality (Fig 1B) was much higher in infected *Ctsl*^*-/-*^ mice (61% versus 34% in *Ctsl*^+/+^ mice receiving identical inocula; *P* = 0.0037).

### Pneumonia Histopathology

**Fig 2:**
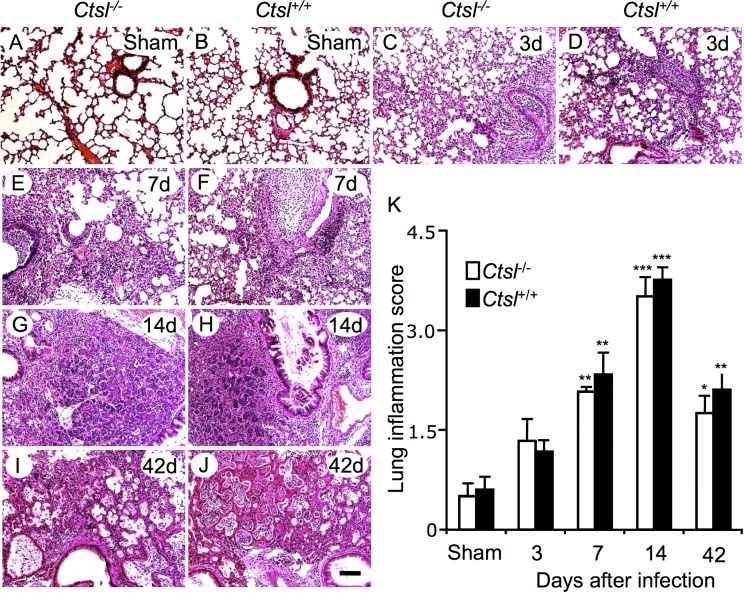
Lung histopathology after influenza A infection. The photomicrographs show representative hematoxylin and eosin-stained sections from *Ctsl*^-/-^ and *Ctsl*^+/+^ mouse lungs from sham-infected animals *(A*, *B)* or from animals sacrificed after 3 *(C*, *D)*, 7 *(E*, *F)*, 14 *(G*, *H)* and 42 *(I*, *J)* days of infection. All images are at the same magnification (scale bar = 100 μm). Regions of metaplasia, bronchiolization and cyst formation in infected lungs are shown in more detail in S1 Fig *(K)* Comparison of pneumonia grades in sham- and virus-infected mice. Responses were compared in *Ctsl*^-/-^ and *Ctsl*^+/+^ mice in lungs harvested at the indicated intervals after infection. The maximum possible score is 4. Data are means ± SEM; *N* = 4–5 mice per group; *P < 0.05, **P < 0.01, and ***P < 0.001 versus grades in sham-infected mice (by *t*-tests).

To evaluate the severity of airway and lung inflammation after infection, lung histopathology and pneumonia scores were compared in *Ctsl*^-/-^ and *Ctsl*^+/+^ mice. All infected mice developed lung infiltrates of inflammatory cells persisting at least 42 days (Fig 2A–2J). Both types of mice developed predominantly mononuclear pneumonia, with associated airway exudates, within 3 days of infection. By 7 and 14 days after infection, both types of surviving mice manifested severe inflammation. By 42 days, pneumonia and mononuclear cell infiltrates remained severe but were decreasing. Histopathological scoring, as quantified in Fig 2K, revealed that pneumonia severity peaked at 14 days in surviving mice and then decreased, without significant differences between infected *Ctsl*^-/-^ and *Ctsl*^+/+^ mice.

### Influenza A-induced Epithelial Metaplasia and Fibrotic Remodeling

Analysis of tissues harvested 3 and 7 days after infection revealed (in addition to luminal and peribronchial mononuclear cell infiltration already described), bronchial and alveolar epithelial necrosis and loss of lining cells (Fig 2), which was patchy and contrasted strikingly with specimens from sham-infected mice. By 14 days after initial infection, tissues manifested focal epithelial metaplasia of airways, as well as alveolar bronchiolization in lung sections derived from both genotypes of surviving mice, with persistent patches of largely mononuclear inflammation (Fig 2 and S1 Fig). The areas of bronchiolization, which appeared to represent coverage by a monolayer of non-ciliated, cuboidal Clara-like cells, tended to produce tumor-like “blooms” sharply demarcated from adjacent normal lung (S2 Fig). These appear to correspond to the keratin 5-positive “pods” observed by Vaughan, Kumar, and colleagues in H1N1-infected mice [19, 21]. At 42 days, this process appeared to have evolved by desquamation of regenerated epithelium, leaving cystic spaces that are likely incapable of gas transfer (Fig 2 and S1 Fig). However, some regions of lung parenchyma were largely intact and unaffected in mice surviving infection. Masson’s trichrome staining revealed fibrosis in some of the affected regions at 42 days. However, no differences in collagen deposition in airway walls or alveolar interstitium were observed between infected *Ctsl*^+/+^ and *Ctsl*^-/-^ mice, as shown in S3 Fig. These results reveal that influenza leads to lung remodeling that is present after immunohistochemical evidence of active infection has disappeared.

### Viral Burden and Persistence

**Fig 3:**
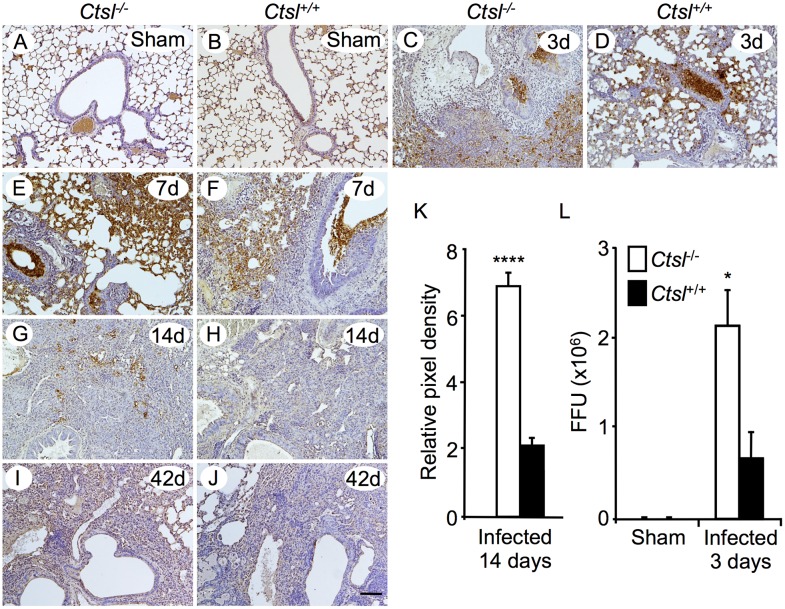
Detection of influenza A in infected *Ctsl*^-/-^ and *Ctsl*^+/+^ lung. Influenza virus was sought by immunohistochemical staining of lung sections from sham-infected animals *(A*, *B)* and from mice 3 *(C*, *D)*, 7 *(E*, *F)*, 14 *(G*, *H)* and 42 *(I*, *J)* days after initial infection. *N* = 3–5 mice per group; scale bar = 100 μm. *(K)* Relative intensity of staining for H1N1 protein was compared by integrating brown pixel density in random fields of lung sections from *Ctsl*^-/-^ and *Ctsl*^+/+^mice 14 days after infection; ****P < 0.0001 by *t*-test. *(L)* Intact viral burden was assessed by measuring influenza A FFU in lung homogenates from *Ctsl*^-/-^ and *Ctsl*^+/+^ mice 3 days after intranasal inoculation with live influenza A or with PBS alone (sham infection). Viral burden is expressed as total FFU in lung homogenates. *N* = 3–5; *P < 0.05 versus FFU in infected *Ctsl*^+/+^ mice by *t*-test. No FFU were detected in lungs of sham-infected mice. Data in *K* and *L* are means ± SEM.

To localize lung tissue sites of influenza A infection and to compare persistence of pathogen in *Ctsl*^-/-^ and *Ctsl*^+/+^ mice, virus was detected immunohistochemically in lung tissue sections incubated with anti-influenza A antibody. Analysis of sections obtained from lungs harvested at intervals after infection revealed that viral antigen persisted in lung and airway epithelium for up to 14 days in *Ctsl*^-/-^ mice (Fig 3A–3J). However, greater viral burden was detected in *Ctsl*^*-/-*^ lung than in *Ctsl*^*+/+*^ lungs at 14 days, as quantified by analysis of pixel coloration corresponding to viral protein immunoreactivity in microscopic fields Fig 3K. No immunoreactivity was detected in either group of mice 42 days after infection. These data suggest that infection lasts longer in *Ctsl*^-/-^ mice. To explore viral expansion in infected lung and to test whether CTSL deficiency impairs anti-viral defenses, influenza A FFUs were assessed in lung extracts. Influenza A FFUs increased 5360-fold in *Ctsl*^-/-^ mice relative to the 400 FFU in the infecting inoculum, compared to a 1680-fold increase in *Ctsl*^+/+^ lungs. Thus, as shown in Fig 3L, *Ctsl*^-/-^ lung extracts had 3.19-fold more activatable influenza A virus than did *Ctsl*^+/+^ lung extracts 3 days after infection. These data suggest that *Ctsl*^-/-^ mice are less effective than *Ctsl*^+/+^ mice in limiting viral replication early in the course of infection.

### Influenza-induced Changes in Immune Cell Populations in Lung and Spleen

**Fig 4:**
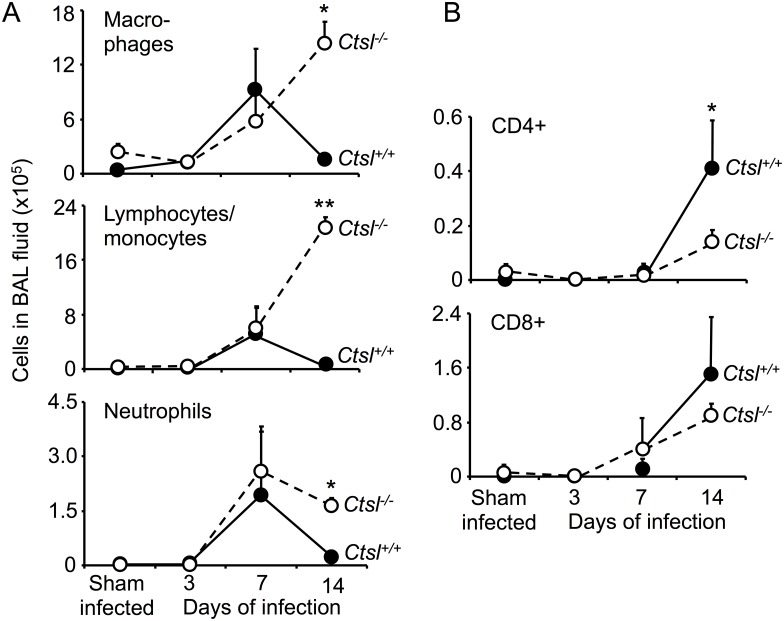
Effect of influenza on luminal immune cell populations. BAL fluid was collected from sham-infected *Ctsl*^-/-^ and *Ctsl*^+/+^ mice or mice infected with influenza A for 3, 7 or 14 days. Panel *A* shows results of analysis of cells identified in BAL fluid by light microscopy. *B* shows results of further analysis by flow cytometry quantifying the proportion of cells expressing CD4 or CD8 within the mononuclear population. Data shown are total cells of the indicated category in BAL fluid (means ± SEM); *N* = 3–5 mice per group; *P < 0.05 and **P < 0.01 as determined by one-way ANOVA.

**Fig 5:**
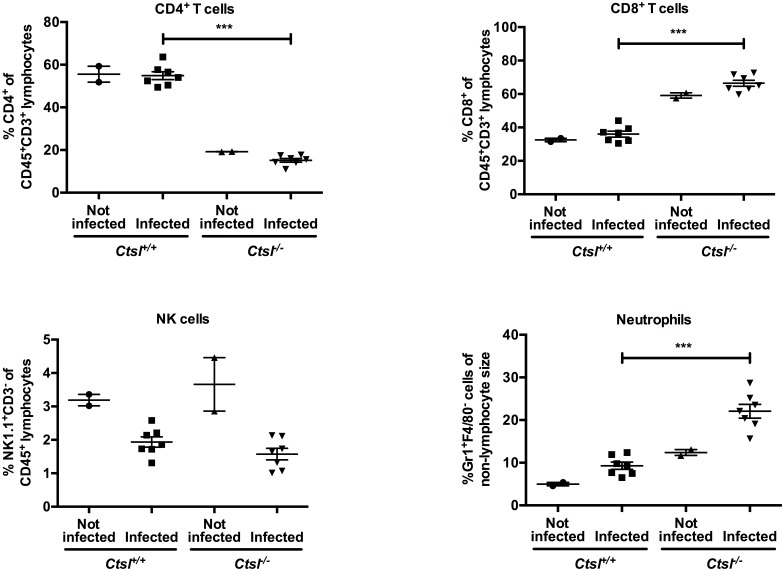
Comparison of splenocyte populations in *Ctsl*^-/-^ and *Ctsl*^+/+^ mice. Spleens were harvested from naïve (not infected) mice and from mice 3 days after nasal inoculation with influenza A virus. CD4+ and CD8+ T cells are a percentage of the CD3+ subset of CD45+ cells. NK cells are the NK1.1+ subset of CD45+ cells. Neutrophils are defined as Gr1+ F4/80- cells and shown as a percentage of cells in the non-lymphocyte gate. *N* = 2 mice in each naive group and 7 mice in each infected group; *** *P* < 0.001, comparing infected groups by *t*-test.

To assess lumen-specific lung inflammation when inflammatory cell infiltrates were evident on histological examination of lungs, BAL fluid cells were assessed in *Ctsl*^-/-^ and *Ctsl*^+/+^ mice 3, 7 and 14 days after infection and in sham-infected mice. In sham-infected animals, total cells in BAL fluid and cytological differential profiles were similar in *Ctsl*^-/-^ and *Ctsl*^+/+^ mice, with neutrophils being uncommon. As shown in Fig 4, inflammatory cells in BAL fluid increased after infection in both types of mice, most dramatically at the 7- and 14-day time points. BAL retrieved more macrophages, lymphocytes/monocytes, and neutrophils, but fewer CD4+ cells from infected *Ctsl*^-/-^ than from *Ctsl*^+/+^ mice at 14 days. There was no significant difference in CD8+ cells in infected *Ctsl*^-/-^ and *Ctsl*^+/+^ mice. In further studies focused on the 3-day time point, when the deaths were starting to occur in both genotypes of infected mice, characterization and quantitation of subsets of inflammatory cells in BAL fluid by flow cytometry (S4 Fig) and splenocyte preparations (Fig 5) revealed modestly higher percentages of splenic neutrophils in infected *Ctsl*^-/-^ mice in comparison to infected *Ctsl*^+/+^ mice. The most conspicuous difference was in CD4+ cells, which were profoundly deficient in *Ctsl*^*-/-*^ mice at baseline and after 3 days of infection, with no significant difference in CD8+ cells.

### Production of Influenza A-directed Antibodies

**Fig 6:**
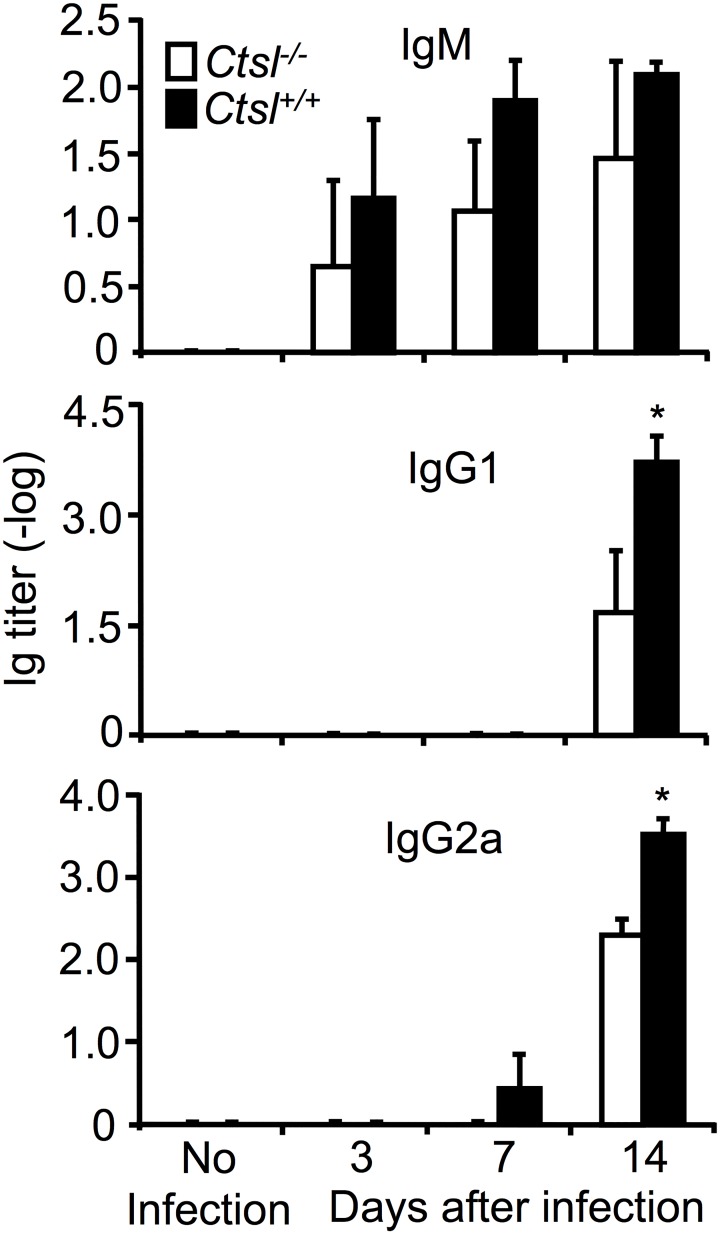
Influenza A-specific antibody titers in serum. Mice were infected 3, 7 or 14 days, as indicated. Data are means ± SEM of log-transformed titers; *N* = 4–5 mice per group; * *P* < 0.05 (infected *Ctsl*^-/-^ versus infected *Ctsl*^+/+^) by one-way ANOVA.

Anti-influenza A IgM titers were lower in infected *Ctsl*^-/-^ mice than in infected *Ctsl*^+/+^ mice at all time points (Fig 6), but the differences were not significant (*P* = 0.61, 0.24 and 0.44 for days 3, 7 and 14, respectively). No titers were detected in influenza A-naive mice, supporting the specificity of the antibodies for influenza A. Anti-influenza A IgG1, which was first detected 14 days after infection, was significantly lower in *Ctsl*^-/-^ mice than in *Ctsl*^+/+^ mice. Anti-influenza A IgG2a was detected later after infection in *Ctsl*^-/-^ mice than in *Ctsl*^+/+^ mice, with titers being undetectable at day 7 and significantly lower in *Ctsl*^-/-^ mice than in *Ctsl*^+/+^ mice at day 14. Anti-influenza A IgA and IgE were not detectable at any time point. These findings suggest that humoral responses to influenza A in *Ctsl*^-/-^ mice are impaired.

### Influenza-induced Changes in Cytokines and Chemokines

**Fig 7:**
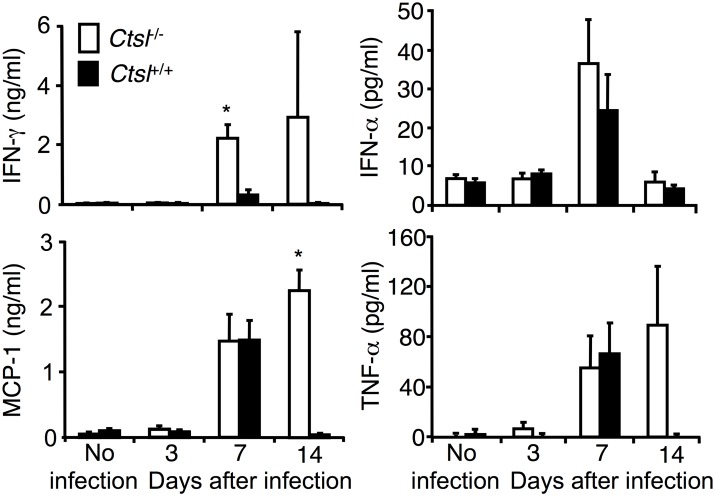
Effect of influenza on BAL fluid levels of IFN-γ, IFN-α, TNF-α, and MCP-1 in *Ctsl*^-/-^ and *Ctsl*^+/+^ mice. Levels were quantified by ELISA in BAL fluid obtained 3, 7 and 14 days after initial infection. Data are means ± SEM; *N* = 4–5; *P < 0.05 (infected *Ctsl*^-/-^ versus infected *Ctsl*^+/+^ mice), by one-way ANOVA.

To assess whether pulmonary responses to influenza A in *Ctsl*^-/-^ mice differ from those of *Ctsl*^+/+^ mice, we compared BAL fluid and/or lung levels of selected proteins. BAL fluid levels of IFN-α, IFN-γ, TNF-α, IL-6, MCP-1 and MIP-2 increased following infection, with levels of IFN-γ and MCP-1 being significantly higher in *Ctsl*^-/-^ mice than in *Ctsl*^+/+^ mice (at days 7 and 14, respectively; Fig 7). Lung tissue levels of these cytokines and chemokines increased slightly after infection but no significant differences were found between *Ctsl*^-/-^ and *Ctsl*^+/+^ mice (data not shown). Thus, these data do not suggest that deficient production of the measured cytokines and chemokines are a basis for the observed differences in responses to influenza A in *Ctsl*^-/-^ versus *Ctsl*^+/+^ mice.

## Discussion

Previous reports suggested that CTSL, by cleaving pathogen-associated proteins, helps Ebola, Hendra and SARS viruses to enter cells, thereby advancing infection [6–8, 22]. CTSL also allows a non-enveloped reovirus to enter cells by digesting outer capsid proteins to form infectious subvirions [23]. In the example of Ebola virus, although certain cathepsins activate some forms of the virus in cell culture, mice genetically deficient in cathepsins are not protected from infection in vivo [24], revealing that viral activation data generated in cell culture do not necessarily predict in vivo consequences. In this regard, the present study does not rule out the possibility that CTSL can activate influenza orthomyxoviruses, which require processing by host proteases to become infectious [11]. However, the findings suggest that CTSL’s dominant contribution is defense against infection, and that its presence in mice limits respiratory viral burden and improves likelihood of surviving infection.

The higher mortality in infected *Ctsl*^-/-^ mice is likely to be related to the observed higher virus burden early after infection as well as the persistence of virus in later stages of infection, with consequently more severe and sustained damage to lung and airways. Although histopathological responses to infection were similar in *Ctsl*^-/-^ and *Ctsl*^+/+^ mice at intervals after infection, as reflected in the inflammation scores shown in Fig 2, dropout of mice dying from infection, via a survivor effect, may have caused underestimation of the severity of inflammation in the *Ctsl*^-/-^ cohort, which experienced greater mortality. Ultimately, poorer outcomes in *Ctsl*^-/-^ mice can be attributed to relative defects in innate immune and pathogen-specific adaptive responses, as suggested by lower titers of IgG in infected *Ctsl*^-/-^ mice relative to those in infected *Ctsl*^+/+^ mice. Given that some of the cited prior work suggesting that CTSL enhances non-influenza viral infectivity is largely based on observations in cultured cells, it is possible that in vivo infections by these or related viruses in *Ctsl*^-/-^ mice would be poorly tolerated due to immune deficits, notwithstanding any reductions in direct viral activation attributable to CTSL’s absence.

CTSL is required for degradation of the invariant chain in cortical thymic epithelial cells, such that *Ctsl*^*-/-*^ mice have decreased positive selection of developing CD4+ T cells. CTSL deficiency also alters the pool of peptides generated for MHC class II presentation. Thus, both quantitative and qualitative defects in the CD4+ T cell repertoire, as reflected by the profoundly depressed proportions of CD4+ splenocytes from *Ctsl*^*-/-*^ mice at baseline and after infection, may result in impairment in early host defenses. Although we did not detect a difference in the early type 1 interferon response, other types of impairment may have been present. For example, differences in nucleic acid-sensing surface proteins like toll-like receptor 7, which contributes to innate recognition of influenza virus RNA and cytokine production by infected macrophages [25] and is activated in part by CTSL [26], also may contribute to the observed early deficit in control of influenza in *Ctsl*^*-/-*^ mice. In some cells, CTSL contributes to antigen processing prior to presentation with MHC II [27], which could lead to defective cellular as well as primary humoral responses, in addition to the established influence of virus-specific CD4+ T cell responses on memory responses and long-term protective immunity [28]. The profoundly depressed proportions of CD4+ splenocytes from *Ctsl*^*-/-*^ mice at baseline and after infection may reflect generalized deficiency in development of CD4+ lymphocytes, with consequent impairment in early host defense. Such defects could include early responses involving innate, heterotypic, or “pre-existing” virus-specific CD4+ cells, which appear to protect humans from severe influenza in the absence of detectable pathogen-specific antibodies [29], possibly by activating the recently recognized cytotoxic potential of CD4+ cells acting on MHC II-expressing epithelial cells [30]. Deficits of CD4+ T cells would also tend to limit their ability to augment killing efficacy of macrophages.

*Ctsl*^-/-^ mice have fewer CD4+ T cells [9] and have defective pathogen-specific T lymphocyte responses to some antigens, as shown, for example, in our studies of *Mycoplasma pulmonis* infection in *Ctsl*^-/-^ mice [10]. However, in the mycoplasma study, no major difference between *Ctsl*^+/+^ and *Ctsl*^-/-^ mice in humoral responses to mycoplasma antigens was observed, as manifested by titers of antibodies in serum. Thus, mycoplasma and influenza pathogens may differ in the manner in which they provoke a humoral response. This may relate to *M*. *pulmonis* being an extracellular pathogen, whereas influenza A infects, kills and lyses target cells. Another overt difference between these two pathogens in mice is the primary nature of the evoked inflammation, which is largely neutrophilic for mycoplasma [10] and largely monocytic/lymphocytic for influenza A (this study). Differences in the inflammatory response could be associated with differences in modes of antigen processing and presentation, in turn yielding differences in cellular and humoral adaptive responses.

The prominent “blooms” of bronchiolization observed adjacent to normal lung in both types of mice by two weeks of infection were similar to histological changes reported in lungs of some autopsied human victims of the 1918 pandemic of influenza A/H1N1 [31]. However, many fatal cases of influenza in 1918 were associated with secondary bacterial pneumonia associated dense infiltration of lung parenchyma with neutrophils, which was not seen in our influenza-infected mice. Although the number of neutrophils retrievable by BAL increased in our infected mice and were higher at the 14-day time point in *Ctsl*^-/-^ mice than in *Ctsl*^+/+^ mice, neutrophils remained a small fraction of total leukocytes at all examined intervals after infection. Whether this represents fundamental differences between humans and mice in response to influenza A infection, differences in viral strains, a survivor effect (because human samples were exclusively obtained from individuals dying after infection), relative protection from bacterial infection by mice housed in barrier facilities, or a combination of these factors remains to be determined. In any case, the histopathology of lung parenchyma 6 weeks after infection suggests that some “bronchiolized” areas naturally evolve during the process of epithelial replacement and repair into cystic air spaces sometimes with surrounding fibrosis. These areas do not represent a return to normal alveoli, are likely non-functional, and may represent permanent damage to gas-exchanging surface of the lung, as may also occur in humans with severe influenza pneumonia and respiratory distress.

Overall, these results suggest a critical role for CTSL in containing airway influenza A infection in mice, in which influenza causes enduring structural changes to alveoli and small airways. Unlike its effect on some viruses, CTSL does not seem to augment influenza virus infectivity, as reflected by comparing viral titers and tissue viral immunoreactivity in *Ctsl*^-/-^ and *Ctsl*^+/+^ mice. However, *Ctsl*^-/-^ mice have defective immune responses, which may contribute to higher mortality after influenza compared to mortality in *Ctsl*^+/+^ mice. These findings also suggest that targeting CTSL for pharmacological inhibition as part of an anti-inflammatory, immunosuppressive or anti-viral strategy, such as suggested for SARS coronavirus, carries a potential risk of impairing host defense against orthomyxoviruses like influenza A.

## Supporting Information

S1 Fig Evolution of lung remodeling after infection. The photomicrographs are high-power views of hematoxylin and eosin-stained sections from *Ctsl*^-/-^ and *Ctsl*^+/+^ mouse lungs harvested 14 or 42 days after infection with influenza virus as indicated. Arrowheads in *A/B* and *C/D* show regions of lung featuring epithelial metaplasia and bronchiolization, respectively. Arrowheads in E/F show later-appearing cystic structures containing cell-rich debris, possibly from sloughed epithelium mixed with inflammatory cells. Scale bar = 60 μm.
(TIFF)

S2 Fig Low-power view of “blooms” of remodeling after influenza A infection. Photomicrographs were taken of sections of *Ctsl*^-/-^ (*A*) and *Ctsl*^+/+^ (*B*) mouse lung harvested 14 days after infection. Scale bar = 600 μm.
(TIFF)

S3 Fig Fibrosis in influenza-infected lungs. Tissues were harvested from sham-infected lungs *(A*, *B)* and from lungs 14 *(C*, *D)* and 42 *(E*, *F)* days after influenza A inoculation. Fibrotic areas in these Masson’s trichrome-stained tissue sections are blue-green.
(TIFF)

S4 Fig Flow cytometry of cells from BAL fluid. CD4 cells were counted as the number of CD45- and CD3-positive lymphocytes in a BAL fluid sample that were CD4-positive. CD8 cells were the number of CD45- and CD3-positive lymphocytes that were CD8-positive. NK cells were CD45-positive CD3-negative lymphocytes that were NK1.1-positive. Neutrophils were F/80-negative non-lymphocytes that were Gr-1-positive. Each data point represents measurement from lavage fluid from one mouse. Infected mice had been inoculated with influenza A 3 days prior to BAL. No significant differences were detected by *t*-test between infected groups at this time point.
(TIFF)
