# Supplementary figures and images for: Cathepsin L Helps to Defend Mice from Infection with Influenza A
Source: PLoS One. 2016 Oct 7;11(10):e0164501. doi: 10.1371/journal.pone.0164501 (PMC5055332; doi:10.1371/journal.pone.0164501)

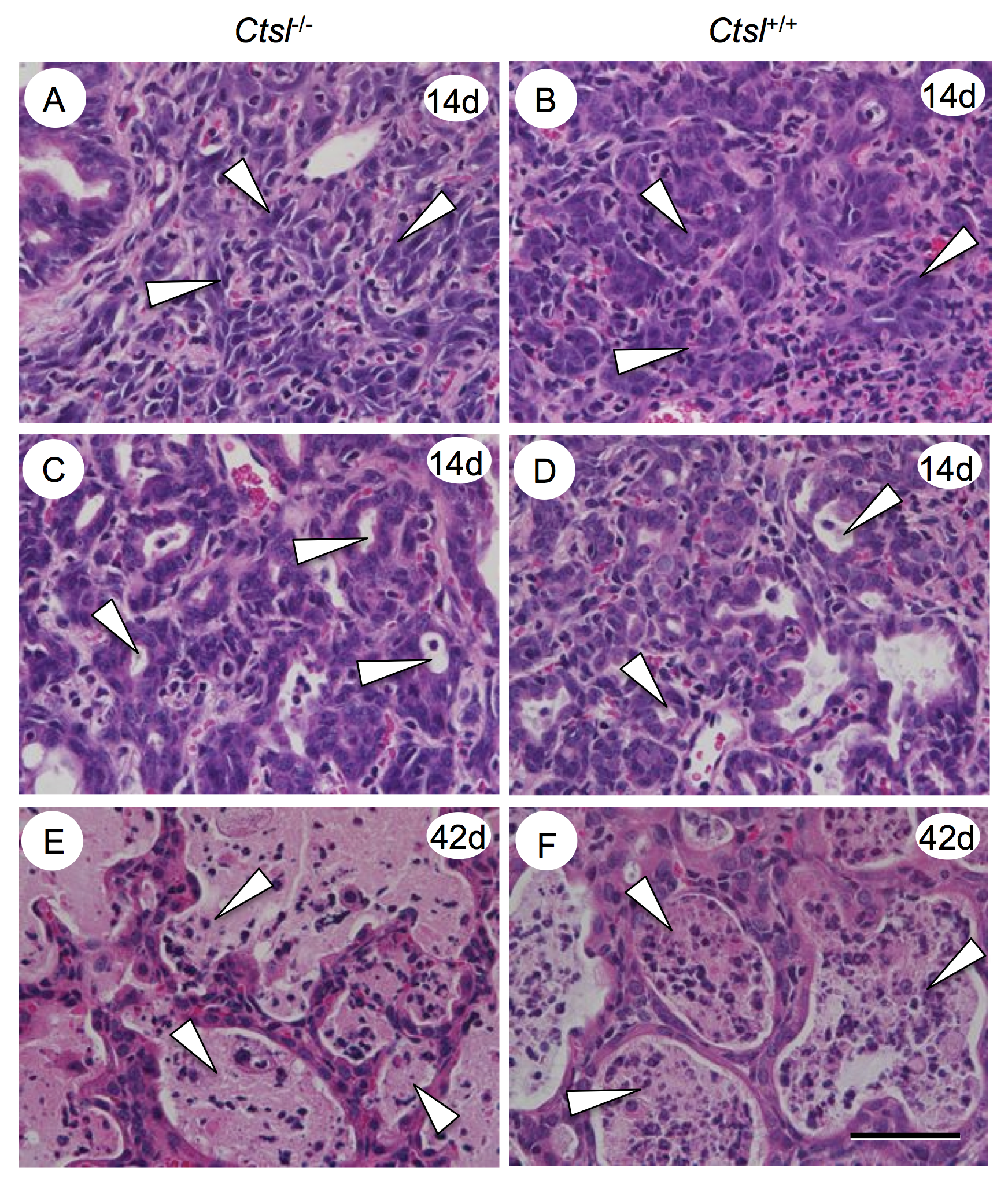

Supplement: S1 Fig — The photomicrographs are high-power views of hematoxylin and eosin-stained sections from Ctsl-/- and Ctsl+/+ mouse lungs harvested 14 or 42 days after infection with influenza virus as indicated. Arrowheads in A/B and C/D show regions of lung featuring epithelial metaplasia and bronchiolization, respectively. Arrowheads in E/F show later-appearing cystic structures containing cell-rich debris, possibly from sloughed epithelium mixed with inflammatory cells. Scale bar = 60 μm. (TIFF) [file pone.0164501.s001.tiff]

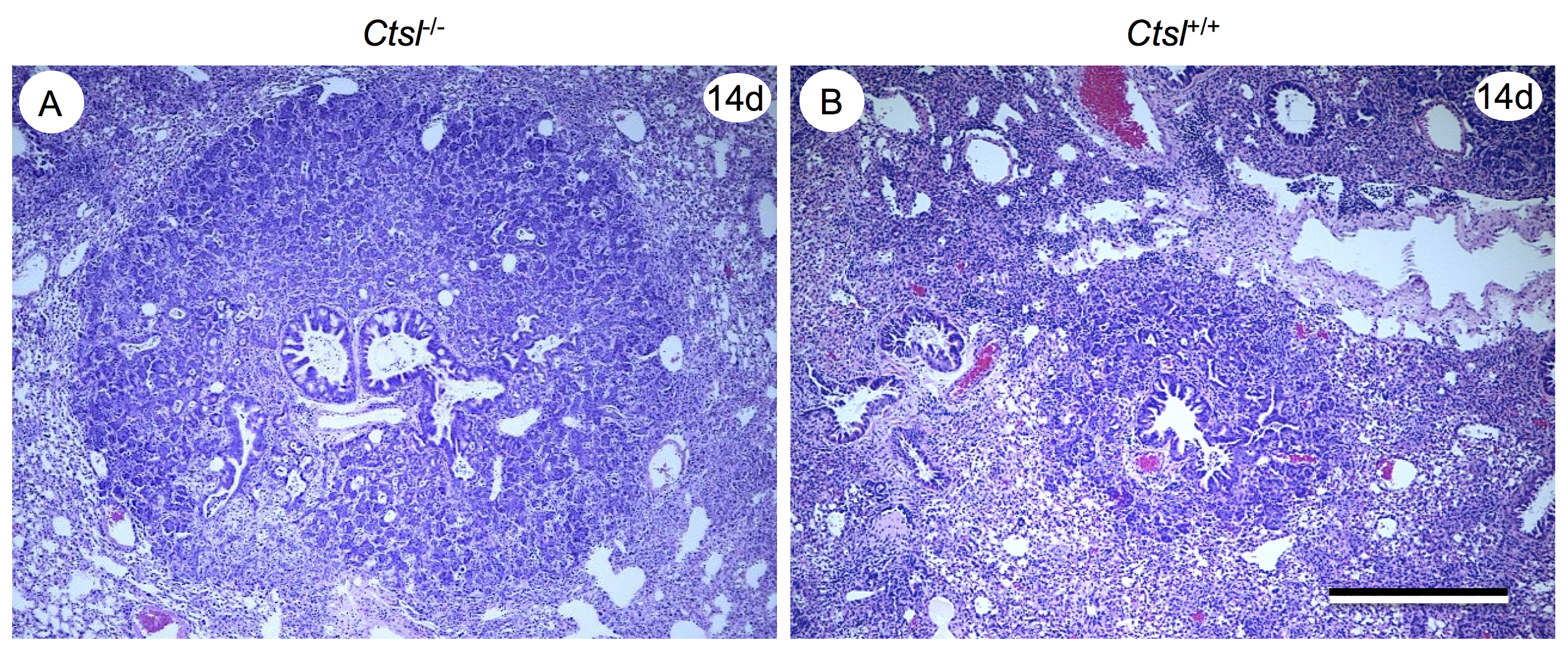

Supplement: S2 Fig — Photomicrographs were taken of sections of Ctsl-/- (A) and Ctsl+/+ (B) mouse lung harvested 14 days after infection. Scale bar = 600 μm. (TIFF) [file pone.0164501.s002.tiff]

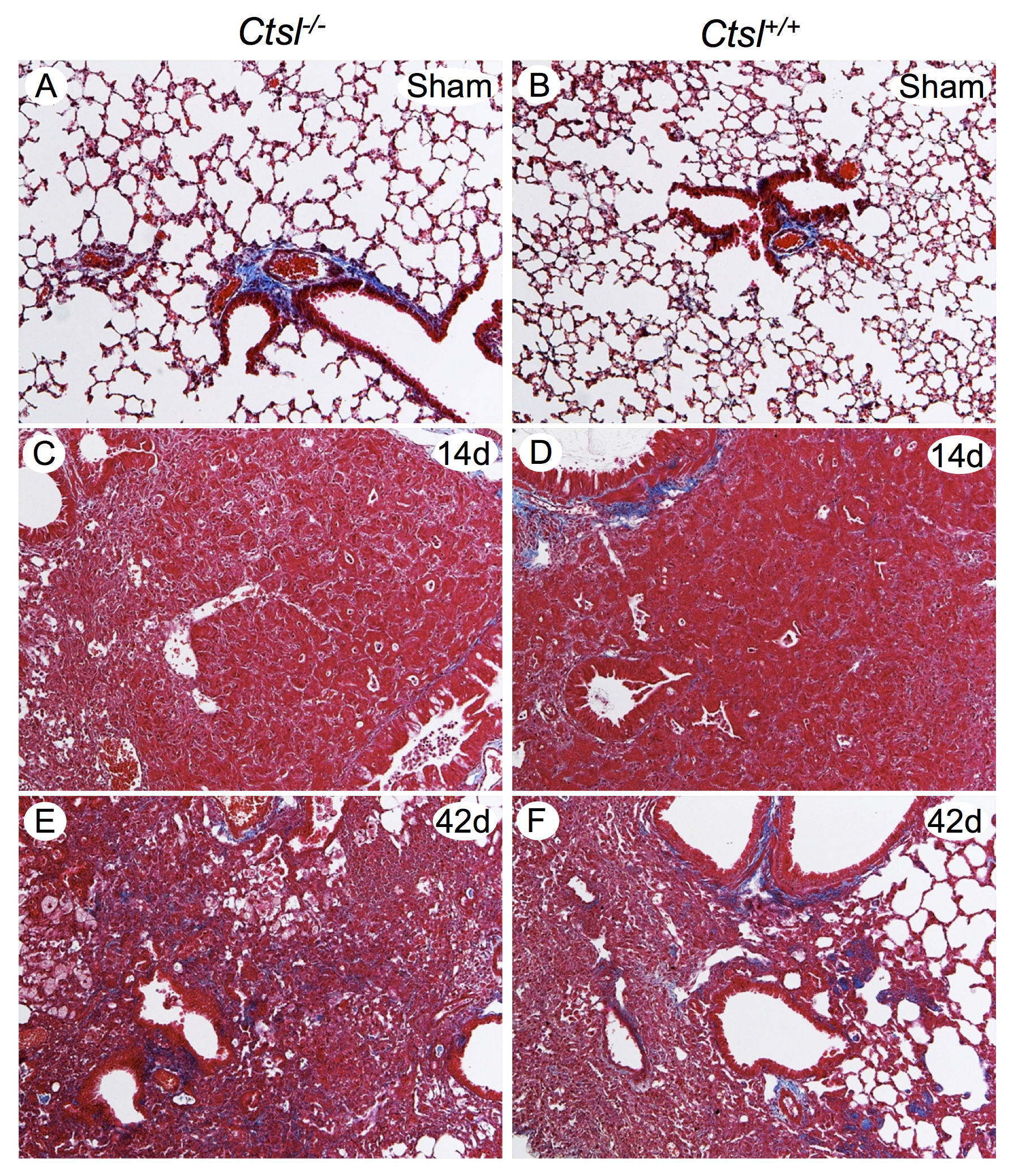

Supplement: S3 Fig — Tissues were harvested from sham-infected lungs (A, B) and from lungs 14 (C, D) and 42 (E, F) days after influenza A inoculation. Fibrotic areas in these Masson’s trichrome-stained tissue sections are blue-green. (TIFF) [file pone.0164501.s003.tiff]

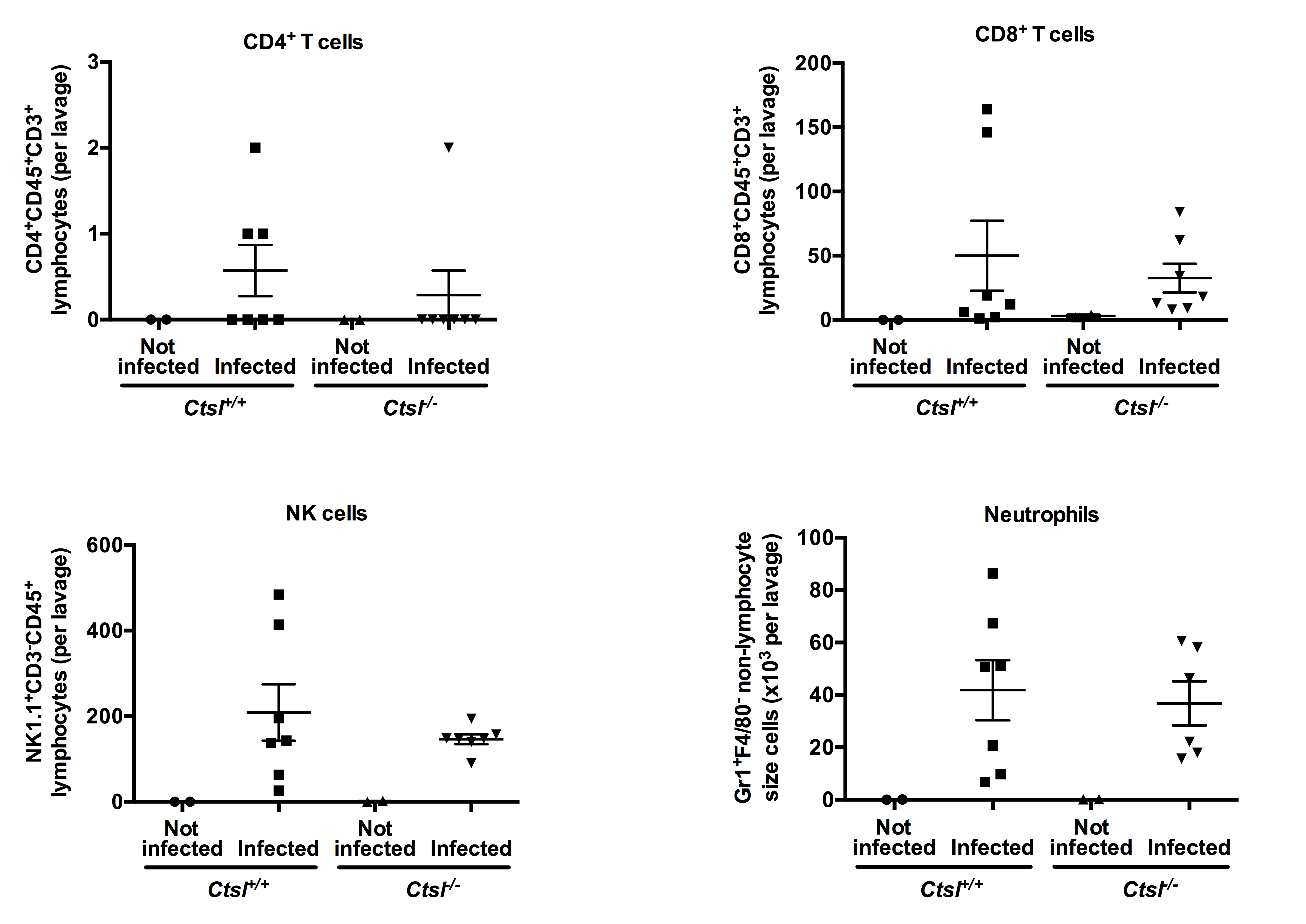

Supplement: S4 Fig — CD4 cells were counted as the number of CD45- and CD3-positive lymphocytes in a BAL fluid sample that were CD4-positive. CD8 cells were the number of CD45- and CD3-positive lymphocytes that were CD8-positive. NK cells were CD45-positive CD3-negative lymphocytes that were NK1.1-positive. Neutrophils were F/80-negative non-lymphocytes that were Gr-1-positive. Each data point represents measurement from lavage fluid from one mouse. Infected mice had been inoculated with influenza A 3 days prior to BAL. No significant differences were detected by t-test between infected groups at this time point. (TIFF) [file pone.0164501.s004.tiff]
